# Child development assessment tools in low-income and middle-income countries: how can we use them more appropriately?

**DOI:** 10.1136/archdischild-2014-308114

**Published:** 2015-03-30

**Authors:** Saraswathy Sabanathan, Bridget Wills, Melissa Gladstone

**Affiliations:** 1 Oxford University Clinical Research Unit, Wellcome Trust Major Overseas Programme, Hospital for Tropical Diseases, Ho Chi Minh City, Viet Nam; 2 Nuffield Department of Medicine, Centre for Tropical Medicine, University of Oxford, Oxford, UK; 3 Department of Women and Children's Health, Institute of Translational Medicine, University of Liverpool, Alder Hey Children's NHS Foundation Trust, Liverpool, UK

**Keywords:** Neurodisability, Neurodevelopment, Outcomes research

## Abstract

Global emphasis has shifted beyond reducing child survival rates to improving health and developmental trajectories in childhood. Optimum early childhood experience is believed to allow children to benefit fully from educational opportunities resulting in improved human capital. Investment in early childhood initiatives in low-income and middle-income countries (LMICs) is increasing. These initiatives use early childhood developmental assessment tools (CDATs) as outcome measures. CDATs are also key measures in the evaluation of programmatic health initiatives in LMICs, influencing public health policy. Interpretation of CDAT outcomes requires understanding of their structure and psychometric properties. This article reviews the structure and main methods of CDAT development with specific considerations when applied in LMICs.

## Introduction

It is estimated that up to 200 million children fail to reach their developmental potential in low-income and middle-income countries (LMICs).^1^ This statistic underpins recent calls for urgent global action to improve early child development in the first 5 years of life.^2^ Poverty, malnutrition, recurrent and/or chronic infectious diseases plus inadequate cognitive stimulation, can all have lifelong consequences on development.^3–5^ Economic modelling studies demonstrate how early interventions and investment in disadvantaged children are effective in improving child development and cost-effective in improving the ‘human capital’ potential of individuals.^6^ Investment in early child development initiatives in LMICs is burgeoning and with this, prioritisation of global health research into interventions that have maximum impact on child development. Specific child development assessment tools (CDATs) are recommended by major research funding bodies as appropriate tools to evaluate child development interventions, despite limited evidence of the nature of the CDATs’ relationship with quality of life measures and later potential human capital.^7^ ^8^ Since no CDAT is suitable for all populations, a variety of tools have been developed worldwide.^9^ Typically, knowledge of the structure, psychometric properties, applicability and limitations of CDAT is restricted to child development specialists. In practice, health workers from various backgrounds often administer and interpret these tests. This article describes the structure, main methods of CDAT development and their application, focusing on children less than 5 years of age in LMICs.

## What is a child developmental assessment tool, what do they measure and how are they administered?

Early childhood is a period of rapid physical growth during which an individual acquires a complex set of skills and functional competencies that should facilitate achievement of their potential in life. Weight and height charts were developed on the premise that children from similar ethnic backgrounds follow similar growth trajectories. In a comparable fashion, acquisition of skills during early childhood is also expected to follow a set trajectory. For growth and development we know that it is best to intervene promptly in order to maintain a healthy trajectory.^10–12^ US legislative changes incorporated in the Individuals with Disabilities Education Act 2004 specify the use of standardised CDATs to evaluate the following developmental domains; cognitive, language, motor, adaptive and socioemotional, in order to guide diagnostic investigations and interventions (table 1).^13^ ^14^

**Table 1:** Developmental domains

Developmental domains and subdomains	Description of domain
Cognitive^39^ ^40^	Strategies and processes children develop to interpret and respond to their environment and experiences including; memory (ability to encode, retain and recall information over time) attention (the ability to choose what to focus on for a sustained period), that influence memory language skills which as the brain develops children acquire and refine language skills. Newborn explores the world by mouthing objects; and later explores the world by imitating actions, manipulating objects and planning two-step strategies to get what he wants. From 2 years, children increase their use of language and start make-believe play. In children aged 3–5 years there is rapid development in information processing (the speed and fluency of response following stimuli), cognitive flexibility (the ability to make and change strategies as required, and to simultaneously process multiple stimuli) and goal setting (the ability to plan strategies in a coherent and efficient order).
Language^41^	
Receptive	Understanding of the spoken word and sentence structure
Expressive	Spoken vocabulary
Motor^42^	
Fine motor	Ability to manipulate small objects
Gross motor	Ability to walk, run and coordinate complex physical activities
Social and emotional^42^ ^43^	The ability to identify and understand one's own feelings and to accurately read and comprehend emotional states in others. Ability to regulate one's own behaviour, to develop empathy for others, and to establish and maintain relationships
Adaptive behaviour^44^	Collection of conceptual, social and practical skills that have been learned by people in order to function in their everyday lives

Each domain of development is assessed by the child's ability to carry out a series of increasingly complex activities that are thought to reflect the expected level of development at a particular age. The developmental domains evaluated by CDAT do not function as discrete entities but influence each other—for example, to successfully complete a jigsaw puzzle requires a combination of cognitive and fine motor skills, and sufficient understanding of language to process instructions.

Methods to assess child development may include: (A) direct assessment of standardised activities by a trained assessor in a clinical environment, (B) verbal reporting/completion of a questionnaire about the child's abilities by parents or teachers and (C) unstructured observation by a trained assessor in an environment familiar to the child (eg, home/school). Direct testing in an unfamiliar environment by an unfamiliar adult may restrict a child's engagement and participation in the assessment. Parental reporting, on the other hand, may be affected by recall bias.^8^ Unstructured observation can be difficult to reproduce and interpret. An ideal CDAT would include aspects of all three methods, but with limited financial resources, often one method is chosen.^8^

## Types of assessment tools—screening versus formal assessment?

Screening tools are administered quickly, using a limited sample of items representing a domain and rely on predetermined cut-off points. Screening tools are designed to identify children who may have impairment and require a comprehensive assessment. However, screening tools have poor utility in assessing subtle delays that may have a significant impact on subsequent development. Screening tools are beneficial when used within the context of a development surveillance programme, where there are appropriate norms and known applicability in specific subgroups.^15^

Most formal tools require specialised specific training of assessors. They allow administration of items above or below the age bracket of the child thus allowing the child's strengths and weaknesses to be categorised more accurately. Formal assessments can be administered repeatedly to evaluate changes over time and/or following an intervention.

## Is the tool measuring what it is supposed to—validity and reliability?

Developmental domains are a theoretical concept, typically referred to as ‘constructs’ in the psychometric literature. These cannot be directly measured but are inferred through the child's performance on a number of observed variables (test items). Reliability is the variability of scores obtained by an individual if repeatedly given the same test. There are widely cited levels of acceptable reliability for testing,^16^ however the result should be interpreted in context. For example, retest reliability can be influenced by developmental maturation over the time interval, or training by the caregiver following mistakes seen in the first assessment.^17^ Validity is the accuracy of the score representing the construct of interest.^8^ In addition, screening tools are evaluated for sensitivity, specificity, positive predictive value and negative predictive value. Reliability and validity of CDAT can be evaluated through several methods, summarised in table 2.

**Table 2:** Basic psychometric properties used to evaluate child development assessment tools (CDATs)

Relevance/Importance	Comment	
Reliability		
Internal consistency	Evaluates the similarity of test items assessed in one domain. One measure is split-half reliability, which compares the scores on two halves of a test in a single domain.	High internal consistency suggests that some items are too similar, so no additional information is gained from assessing them. Low internal consistency suggests the items may not be assessing the same domain.
Interobserver	Evaluates variability between different assessors on the same subject	There may be systematic errors, specific to a particular group of assessors, and this parameter may not be generalisable when the tool is used by a different group of assessors.
Intraobserver	Evaluates variability within a single assessor on a single subject	Commonly evaluated by the same assessor scoring video recordings of their own assessments. This is not essential unless there is low interobserver reliability
Validity		
Test-retest	Evaluates variability within the subject (influenced by random factors such as familiarity with items and mood)	Difficult to interpret in early childhood when changes in development occur over a short time. Usually the repeat assessment should be carried out within 2 weeks of the first test.
Content	Experts in the field make consensus agreement on whether the individual item and the range of items adequately sample and represent the domain of interest.	Subjective measure that cannot be used in isolation to evaluate validity.
Criterion	Ideally assessed by comparison to an established ‘gold standard’ test assessing the same construct	Usually ‘gold standard’ tests are not available so the comparison is typically against another recognised test regularly used in the same population and thought to measure the same domain.
Discriminant/convergent	Evaluates expected positive and negative correlations between scores in different domains or between different tests of the same or differing underlying construct.	Scores from two independent tests (eg, one using report method the other a direct test) of one domain should correlate where neither test is considered a ‘gold-standard’. To ensure the test is not overlapping with constructs not of interest, the scores evaluating different constructs should poorly correlate, for example, test scores on ‘fine motor’ should correlate poorly with ‘social emotional’.
Construct	Statistical evaluation to see whether values of observed data fit a theoretical model of the constructs (confirmatory) or to explore a possible model of the ‘underlying traits’ being measured.	Large numbers of assessments are required to evaluate this.

## How to determine a child's performance on the CDAT—the reference population

All assessment tools have a threshold that a child must achieve for the assessor to be confident that there are no current developmental concerns. There are two main ways of setting this threshold, norm referencing and criterion referencing. A norm-referenced test is usually administered in a standardised manner and the individual child's scores in each domain are compared with scores from a large representative sample of children of the same age and sex (normative data). Standard scores allow comparison between scales evaluating the same domain and monitoring of individuals at different ages. By contrast, criterion-referenced tests assess whether a child has acquired a particular skill by a certain age, according to a specific curriculum. Administration may not be standardised, since individual children sometimes require additional instructions or physical aids to complete a particular task but are then able to complete the specified tests. Criterion-referenced tests are often used in screening procedures, for example assessment of reading skills at school entry, or evaluation of an intervention such as a programme of physical therapy. In some cases criterion-based tests have been standardised on large representative populations.

There are concerns that the rapidly changing nature of society means that normative data become outdated very quickly. To counter this, inclusion of a control group is now considered crucial for research studies, even when the normative population is contemporaneous and comes from the same linguistic and cultural background as the study population.

## When to assess development, what does it mean for the future—predictive validity?

CDAT will only give a snapshot of the child at that time point. Traits that have not yet evolved in early childhood clearly cannot be assessed until such time as they might reasonably be expected to be present, and yet impairment within these characteristics may impact subsequent development significantly.^18^

After an assessment, it is important to know the predictive validity of the CDAT score, whether normal or abnormal. Correlations between early childhood performance and school abilities in normal children are variable.^19^ Children with moderate developmental delay, despite the same initial difficulties, have great variability in developmental trajectories, with some children catching up while others exhibit evolving difficulties with age.^20^ Aspects of early development may be predictive of later academic ability. Preschool English language acquisition of vocabulary, grammar and descriptive phrasing predicted school reading ability in a prospective longitudinal US study.20a Research in normal infants suggests that acquisition of efficient inhibitory control and planning aspects of cognitive executive function at 4 years of age are associated with improved acquisition of school mathematical and literacy abilities at 6–7 years of age.^21^ ^22^

There is limited research examining the predictive nature between CDAT and longer-term outcomes in LMICs. Three longitudinal nutritional intervention studies collected CDAT scores in infants from Indonesia and Guatemala.^23^ Scores on 2 monthly assessments correlated poorly in infants less than 24 months, but score correlations increased on assessments carried out between 24 months and 30 months of age. The scores at age 20 months or younger had no predictive value for verbal reasoning and arithmetic scores at school age. Overall, predictive power in comparative US research was similar.^24^ There is still a lack of evidence as to whether the interpretation of group differences in scores due to an intervention or exposure in infancy should be interpreted as predicting anything in terms of later intelligence.^23^

## Approaches and challenges of CDAT development in LMICs

Currently, only a few CDATs are available in LMICs and most are used in research settings. These CDATs typically follow one of the following formats: (A) a standard western CDAT with no adaptations; (B) a western CDAT translated (linguistic equivalence) and/or adapted for the local cultural environment (cultural equivalence); (C) an amalgamation of a number of translated and/or adapted items from several different western CDATs; or (D) a locally developed, culturally specific CDAT consisting of original items designed to be relevant to the population of interest.^25^ The format of CDAT adaptation depends on the aim of the application. Rie *et al* evaluated the impact of HIV on neurodevelopment of children in Kinchasa, Congo. The researchers deliberately used a direct translation of a western CDAT without cultural adaptation to compare between groups in the same setting and then compare between past and future studies in other settings.^26^ This method does not ensure that the tool is measuring the same underlying ability in all settings. Holding *et al*^27^ aimed to facilitate comparisons between different cultural groups in differing countries and needed to measure the same underlying ability in all settings. This was done by cultural modification of a western tool. The Kilifi Developmental Checklist (KDC) was also based on items from a range of CDATs.^28^ The items were chosen based on ease of observing item success, how well the item could differentiate within the population of interest and if the item could be readily described in the local languages. Additional considerations pertaining to LMICs include the prohibitive cost of the license fee to translate or adapt a western CDAT, the level of skill of the assessor and location of assessment. Fernandes *et al*^29^ developed a screening tool for use in high-income countries and LMICs. The team faced the challenge of developing a culture-free tool that was low in cost, could be administered after limited level of assessor training and completed in 30 min. To achieve this, an advisory panel listed project-specific criteria that the CDAT needed to fulfil.

Once items are selected, the first step is pilot studies on a small number of children. These aim to discover the range of responses elicited from the tested population and identify problems with unfamiliar stimulus materials. Prado *et al*^17^ adapted a cognitive test for Indonesian children, who had difficulty identifying a picture of a bunny, so replaced it with a picture of a chicken. Despite modification to the KDC following pilot studies, audit of final study scores found some items could not be successfully completed by at least one child less than 36 months of age.^30^ These data were used to refine and then expand the KDC to create the Kilifi Developmental Inventory.^30^

In some situations, no appropriate tool exists and new test items need to be devised. This requires understanding of the domain being measured and the cultural references of the relevant population, but can still be problematic. The most common method of developing new items is to engage participant representatives of the population in focus groups. Decisions regarding who will participate and how the information will be transcribed and analysed are important factors in the process. Local experts evaluate the suitability of particular items for a target group, and evaluate whether items adequately cover the domain under investigation. Gladstone *et al*^31^ adapted a tool for Malawi but needed to devise new items for the social/emotional domain. Themes for new items were devised from focus groups, and despite good face and content validity, pilot testing results identified that the new items did not perform well when compared in terms of their psychometric properties alongside original items. An iterative process of adaption and testing led to two more draft versions of the Malawi Developmental Assessment Tool before the team successfully developed the final version.^32^

Table 3 presents examples of the basic structure and psychometric properties of several CDATs developed for LMICs.

**Table 3:** Examples of CDATs (children <5 years) used in LMIC countries sourced from published literature

Tool and country	Type of tool and item selection (translation±adaptation)	Reliability	Validity	Comments
	Method of assessment	Internal consistency	Criterion	
	Age	Interobserver	Discriminant	
		Test-retest	Construct
Kilifi Developmental Checklist (KDC) Kenya^28^	Mixture of items from government local screening test, Western tools and LMIC tools developed elsewhere	Excellent*	Items range from poor to good correlation with parental report†	Aimed to be used by low-skilled workers. Identified that secondary school level education was minimal for an assessor.
	Direct and Report	Excellent‡		
	11–109 months	Excellent‡	Done	
Kilifi Developmental Inventory (KDI) Kenya^30^	Assessment based on KDC with additional items from western tools	Excellent*	Good correlation with maternal reports†	Designed to monitor changes over time. Involved parental focus groups so procedures acceptable to community.
	Direct observation	Excellent‡	Sensitive to neurodevelopmental disorders and underweight children.	
	6–35 months	Excellent‡		
Developmental Milestone Checklist Kenya^45^	Screening Items from Western tools	Good*	Very good correlation with KDI†.^30^	Structured interview. Future plans to develop cut-offs using clinical samples. Can be administered by those with little training.
	Report		Stunted children had lower scores.	
	3–24 months	Good‡	Done	
Guide to monitoring child development. Turkey^46^	Screening Core ideas were adapted from three seminal western models of child development.	Excellent*	Excellent agreement with comprehensive evaluation§ Sensitivity 0.88; 95% CI 0.69 to 0.96 Specificity 0.93; 95% CI 0.83 to 0.97	Brief, open-ended precoded interview with parent. No specific cognitive domain questions. May not be appropriate for monitoring ‘at-risk’ populations.
	Report	Good§		
	0–24 months			
Rapid neurodevelopmental assessment RNA 0–2 years Bangladesh^47^	Screening Mixture of neurological examination and neurodevelopment from western tools		Significant differences in scores in children with neurodevelopment impairment	Used in a small high-risk population and identified infants requiring intervention for retinopathy of prematurity. RNA identified additional problems such as impairments in vision and hearing.
	Direct	Excellent§	Detected difference in scores between urban rural children in older age groups	
	0–24 months			
Rapid neurodevelopmental assessment. RNA 2–5 years Bangladesh^48^	Screening Mixture of neurological exam and development assessment from western tools		Sensitivity 80–90% Specificity 60–78%	Comparison with ‘gold-standard’ tests already developed for Bangladesh.
	Direct	Excellent§	Stunted children had lower scores.	
	24–60 months			
Malawi Developmental Assessment Tool. Malawi^32^	Screening Western developmental tool and new items locally devised	Excellent^49^		Good strategy to devise socioemotional items.
	Direct and report	60–80% of items at least fair§	Sensitivity 97% Specificity 82% Sensitive to malnutrition except social developmental domain	
	0–72 months	99–100% scoring at least fair§	Good predictive validity^50^ ^51^	
INTERGROWTH-21st Neurodevelopment Assessment^29^	Screening Items from selection of Western and LMIC tools			Project specific criteria to create a tool for use in children from middle-class and upper class families across low-income, middle-income and high-income settings, carried out by non-specialists. Also evaluates vision, hearing and sleep-wake cycle.
		Direct and report	Good	
		24 months	Excellent	
Systematic approach to assess nutritional influences. Indonesia^17^	Assessment Items from selection of Western tools	Items at least fair*		Clear hypothesis driven tool creation. May not be suitable as a general developmental assessment.
	Direct	At least fair agreement¶	Expected relationship with maternal depression and maternal education	
	22–55 months	Items at least fair†		
Interpretation of statistical tests are listed below.^16^ ^52^ These need to be interpreted in context of application and population.^17^ ^53^				
Levels of κ Levels§, R†, intraclass‡ correlation and α coefficients*.		Level of proposed agreement¶ (%)		Levels of clinical or practical significance
<0.4		<70%		Poor
0.4–0.59		70–79		Fair
0.60–0.74		80–89		Good
0/75–1.00		90–100		Excellent

CDAT, child development assessment tool; LMICs, low-income and middle-income countries.

## What have we learnt from CDATs in LMICs: factors that influence early child development in LMICs

Debate on the influence of nurture versus nature continues, although an increasing body of opinion now considers the early perinatal environment to be as important in determining cognitive ability as an individual's genetic background. Intrauterine environment, nutritional deficiency in infancy, infectious diseases and poverty, all shape the developmental trajectory of a child in a LMIC. What we know about these threats to development come from research using CDAT. Walker *et al*^33^ reviewed the risk and protective factors of early child development that can be modified by interventions in children under the age of 5 years in LMICs, including iron deficiency, malaria and inadequate cognitive stimulation. These three factors illustrate challenges of CDAT interpretation. Three prospective iron supplementation trials unexpectedly found no change in cognitive development measured by an adapted western CDAT.^34^ It was plausible that the CDAT was not sensitive enough to the effects of supplementation of a nutritional deficiency, resulting in a shift to a more hypothesis-driven, focused testing of specific areas of development.^35^ This shift to ensure the CDAT can evaluate hypothesised outcomes based on biological mechanisms was incorporated into developing an appropriate CDAT for severe malaria.^36^ A team in Kilifi adapted a western tool for school-age children and then successfully evaluated its construct validity and sensitivity for discriminating cognitive deficits following severe malaria (high-risk), mild malaria (medium risk) and no previous admission with malaria (low-risk).^27^ There were significant differences in scores between unschooled high-risk children and unschooled low-risk children. There was no significant difference in scores between high-risk and low-risk malaria children who were at school, highlighting the plausible benefit of formalised education in protecting from neurocognitive sequelae in LMIC settings. This finding was put into context by the authors, who reported that the group attending school was not necessarily representative of severe children, since school attendance is limited by economic restraints and parental perception of whether their child would benefit from formal schooling. A child perceived to have cognitive deficits by their parents might not be sent to school. In a setting with a multitude of biological threats to early child development, the addition of poverty will limit the beneficial opportunities of a child's environmental influence on their development. However, there is variability in parental attitudes and behaviours within one socioeconomic class and also variability within the same socioeconomic class between cultures. A South African Home Screening Questionnaire, found mental development was primarily accounted for by the mother's ability to structure the child's environment for learning.36a This was through the provision of play materials and maternal involvement in the child's activities, which were believed to promote development. The authors concluded that the socioeconomic variables evaluated did not solely determine maternal behaviour. Supporting this premise are studies suggesting maternal stimulation programmes within early childhood community-based stimulation interventions that can have long-lasting benefits on cognitive, socioemotional well-being despite adverse socioeconomic environments.^37^ CDAT research will continue to inform the agenda for early intervention, before lifelong trajectories leading to large health and economic inequalities become fixed.

## Conclusions and future directions

Childhood developmental outcomes are firmly on the current global health agenda particularly now that it is time to set the new millennium developmental goals. However the relationship between future employability, quality of life measures and performance on a CDAT before the age of 5 years, is not known. Longitudinal research is needed to evaluate these relationships. In LMICs where early intervention support is limited, robust CDAT construction within culturally acceptable early child development programmes will help explore the relationship with later outcomes and promote the factors which protect against the neurocognitive detrimental effects of adversities faced by children in LMICs.^38^

## Quick checklist for appraising a CDAT

1. Does the CDAT adequately measure all aspects of the domain(s) theoretically affected by a risk factor or intervention?
2. Has the CDAT been shown to be reliable and valid in the population of interest?
3. Is the CDAT sensitive enough in the setting required to identify the changes expected for the risk factor or intervention?
4. Are the number of evaluations and duration of follow-up suitable for evaluating the outcome of interest?
5. In research studies, is there a suitable control group and have potential influences been considered?
